# Single‐cell transcriptomic analysis reveals subtle fluctuations of oocyte cryopreservation on mouse early embryo development

**DOI:** 10.1002/ctm2.70801

**Published:** 2026-09-07

**Authors:** Yixin Ren, Qingyang Zhang, Fanqing Xu, Yao Li, Tianyi Liao, Mengyuan Zhao, Zhiqiang Yan, Xiaohui Zhu, Nan Wang, Peng Yuan, Gary D Smith, Jie Qiao, Jie Yan, Liying Yan, Qilong He

**Affiliations:** ^1^ Department of Obstetrics and Gynecology Center for Reproductive Medicine Peking University Third Hospital Beijing China; ^2^ State Key Laboratory of Female Fertility Promotion Center for Reproductive Medicine Peking University Third Hospital Beijing China; ^3^ National Clinical Research Center for Obstetrics and Gynecology Peking University Third Hospital Beijing China; ^4^ Key Laboratory of Assisted Reproduction Peking University Ministry of Education Beijing China; ^5^ Beijing Key Laboratory of Reproductive Endocrinology and Assisted Reproductive Technology Beijing China; ^6^ Peking‐Tsinghua Center for Life Sciences Peking University Beijing China; ^7^ School of Public Health Shandong University Jinan China; ^8^ Department of Obstetrics and Gynecology University of Michigan Ann Arbor Michigan USA; ^9^ Shandong Center for Disease Control and Prevention Jinan China

**Keywords:** assisted reproductive technology, embryo transcriptome landscape, oocyte cryopreservation, single‐cell RNA sequencing

## Abstract

**Background:**

Oocyte cryopreservation is widely used in assisted reproductive technology, but its effects on embryonic development remain a concern. This study aimed to determine whether oocyte cryopreservation induces transcriptional alterations at the early cleavage and blastocyst stages.

**Methods:**

We performed single‐cell RNA sequencing on mouse Metaphase II oocyte and embryos at early/late 1 cell, early/late 2 cell, morula and blastocyst stages derived from cryopreserved (cryo) and fresh (fresh) oocytes. Transcriptomic profiles were compared between the two groups.

**Results:**

Global transcriptomes were highly similar between cryo and fresh blastocysts. Oocyte cryopreservation did not alter the sex ratio or the developmental progression through preimplantation development. In blastocysts, cryopreservation affected only a few individual genes. In both male and female blastocysts, although oocyte cryopreservation increased the proportion of low *Xist*‐expressing cells, it did not disrupt X‐linked gene dosage. In the inner cell mass (ICM) of blastocysts, a negative correlation between X‐linked gene expression and cell cycle progression was identified, and this negative correlation was more pronounced in trophectoderm (TE) cells. Overall, this stage‐dependent coordination remained unperturbed by cryopreservation. CellChat analysis revealed that the signalling interaction strength between ICM and TE also showed no significant changes in the cryo group.

**Conclusions:**

Oocyte cryopreservation does not severely disrupt overall transcriptional integrity, developmental potency and X‐chromosome dosage compensation of embryos, but it may cause subtle molecular changes. These findings highlight the need for continued refinement of cryopreservation methods and further investigation into the sub‐acute safety of assisted reproductive technology procedures.

## Key points

1. Single‐cell transcriptomics maps the continuous developmental dynamics from metaphase II oocytes through the blastocyst stage following cryopreservation.
2. Lineage‐specific resolution enables precise molecular evaluation of the inner cell mass and trophectoderm, overcoming previous whole‐embryo technical limitations.
3. The stability of X‐chromosome dosage compensation and its temporal coordination with cell cycle progression are systematically assessed under cryo‐stress.
4. Autocrine and paracrine signaling networks within the blastocyst are profiled to investigate potential cryo‐induced developmental rewiring.

## Graphical abstract

Mature oocytes were subjected to cryopreservation followed by intracytoplasmic sperm injection (ICSI). Embryos at multiple developmental stages were collected, dissociated into single cells and processed for single‐cell library construction and bioinformatics analysis. Cells from the same developmental stage clustered together, whereas no clustering difference was observed between cryopreserved and fresh oocyte‐derived embryos. Remarkably, the intercellular communication dynamics remained largely intact, implying that oocyte cryopreservation exerts minimal impact on normal embryonic signalling.

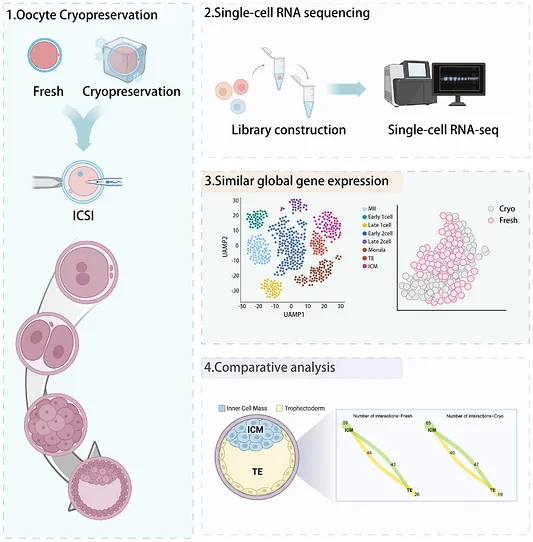

## 1 INTRODUCTION

Oocyte cryopreservation offers women suffering from infertility and other conditions the opportunity to preserve their fertility by storing oocytes for future use. Additionally, cryopreserved oocytes can be utilised for elective fertility preservation related to disease.^1^ The first case of oocyte cryopreservation dates back to the 1980s, when slow freezing was primarily used to prevent oocytes from dehydrating and forming ice crystals during gradual cooling.^2^ Oocyte vitrification, as a more advanced fertility preservation strategy than slow freezing, has been applied in clinic for years.^3^, ^4^, ^5^ As the increase of the born babies from vitrified oocyte, the safety for offspring of this method has been discussed. Studies have demonstrated that vitrified oocytes functionally equivalent to fresh oocytes in terms such as fertilisation, embryonic development and implantation potential.^6^, ^7^, ^8^, ^9^, ^10^, ^11^ As for the clinical outcomes and offspring safety of oocyte cryopreservation, there were also studies. A comparative analysis of data from 1027 children born after frozen oocyte embryo transfer and 1224 children born after fresh oocyte transfer found no increased risk of adverse obstetric and perinatal outcomes in children conceived using vitrified oocytes.^12^ A systematic review included 4159 babies conceived following oocyte vitrification revealed no elevated risk of adverse neonatal outcomes.^13^ A retrospective study of 843 women indicates that both warming cryopreserved oocytes and utilising fresh oocytes for assisted reproductive technology (ART) yield favourable reproductive outcomes (cumulative live birth rates of 41.1% and 48.1%, respectively), with the optimal clinical strategy depending primarily on the woman's age upon return to the clinic.^14^

The mouse model is highly representative in the study of the impact of oocyte cryopreservation on preimplantation development due to its conserved regulatory networks. Although significant species‐specific differences exist, particularly regarding the timing of zygotic genome activation (ZGA) and the mechanisms of X‐chromosome inactivation. In contrast with mice, humans exhibit biallelic *XIST* transcription during the dampening phase, allowing X‐chromosome genes to remain expressed from both alleles in parallel with the progression of dosage compensation.^15^ However, the use of animal model enables a stringently controlled experimental design to uncover the subtle molecular perturbations induced by cryopreservation—analyses that would be ethically and practically unfeasible using human clinical specimens. The mouse is the most widely utilised animal model in studies concerning oocyte cryopreservation and early embryonic development. Due to the temperature change, osmotic stress, cryoprotectant toxicity and/or phase transitions, the negative effects of cryopreservation process to embryo developmental potential were still not clear.^16^ Recent studies have revealed that oocyte cryopreservation might cause physiological and transcriptomic changes.^17^ While blastocyst formation and chromosomal aneuploidy rates remain comparable between fresh and frozen oocytes, cryopreservation may disrupt epigenetic mechanisms. This disruption could alter embryonic gene expression, leading to developmental abnormalities in placental or foetal stages and increasing disease susceptibility in adulthood.^18^ In animals, blastocysts following oocyte cryopreservation may exhibit a diminished methylation profile.^19^, ^20^ Current human data have reported no significant epigenetic alterations following cryopreservation.^21^, ^22^, ^23^ By sequencing technologies, researches have shown that vitrified or slow‐frozen oocytes exhibit transcriptomic changes, with gene expression in human Metaphase II (MII) oocytes altered, including the downregulation of genes related to embryo development, energy metabolism, DNA integrity, cell cycle and this change is more pronounced in slow‐frozen oocytes.^24^, ^25^ These findings present new challenges regarding the impact of cryopreserved oocytes on the health and safety of foetal development in subsequent assisted reproductive technologies. Few studies have reported the global transcriptome effects of oocyte cryopreservation.^26^ Previous studies have mostly been limited to the cryopreserved oocytes themselves; while the expression of specific genes in embryos derived from cryopreserved oocytes was detected using techniques such as real‐time quantitative PCR (RT‐qPCR) and immunofluorescence,^19^, ^26^ the impact on the global transcriptome of blastocysts remains unclear. In mice, a study identified no differentially expressed genes (DEGs) between cryopreservation and control oocytes RNA‐seq.^27^ Conversely, studies detected a variation in gene expression levels specifically in bovine and porcine oocytes.^28^, ^29^, ^30^ Oocyte cryopreservation impairs the translation of maternal spliceosome genes, propagating defects to early embryos that cause widespread RNA alternative splicing abnormalities at the 2‐cell stage.^31^ Starting from the blastocyst stage, embryos undergo distinct cellular differentiation, where the cell fates of the inner cell mass (ICM) and the trophectoderm (TE) are markedly different. Due to technical limitations, previous studies could only detect gene expression in the blastocyst as a whole. However, single‐cell RNA sequencing (scRNA‐seq) technology can distinguish between these two cell types based on the expression of lineage‐specific genes, thereby allowing for a better exploration of the impact of oocyte cryopreservation on embryonic development. Nevertheless, the global transcriptome stability of early embryos from cryopreserved oocytes remains unclear. Therefore, in this study, the global transcriptional effects of mouse oocyte cryopreservation on TE and ICM cells, as well as oocytes, early/late 1 cell embryos and early/late 2 cell embryos, were explored by scRNA‐seq. This study demonstrates that oocyte cryopreservation exerts minimal impact on the global transcriptome and X‐chromosome dosage compensation of mouse embryos, reinforcing the technique's molecular safety.

## 2 MATERIALS AND METHODS

### 2.1 Animals and ethics

The experiment was conducted in strict accordance with the guidelines set by the Ethics Committee of Peking University Third Hospital (ethics approval number: A2021315). Seven‐week‐old female C57BL/6J mice and age‐matched male 129/SvEvBrd mice were purchased from Beijing Huafu Kang Biotechnology Co., Ltd. Female mice were housed in groups of four per cage, while male mice were housed individually. All experimental animals were kept in a clean‐grade animal facility at the Peking University Health Science Center, where they were provided with adequate growth and reproduction feed (purchased from Beijing Keao Xieli Feed Co., Ltd.) and sterile drinking water. The temperature in the housing environment was maintained at 22 ± 2°C, with a 12‐h light/dark cycle. Newly acquired mice were allowed to acclimate to the animal facility for 1 week before the downstream experiments commenced. All mice were anaesthetised using sodium pentobarbital before cervical dislocation was performed. Unless otherwise specified, all reagents used in this study were purchased from Sigma–Aldrich Co., Ltd.

### 2.2 Preparation of oocyte cryopreservation solution

Equilibrium medium (EM) used for processing mouse oocytes contains 7.5% (v/v) ethylene glycol (EG), 7.5% (v/v) dimethyl sulfoxide (DMSO) and 20% (v/v) foetal bovine serum (FBS), dissolved in Dulbecco's phosphate‐buffered saline (DPBS). The vitrification medium used for processing mouse oocytes contains 15% (v/v) EG, 15% (v/v) DMSO,.5 M sucrose and 20% (v/v) FBS, dissolved in DPBS.

### 2.3 Oocyte collection, cryopreservation and thawing

For superovulation induction, PMSG (5–10 IU per mouse) was administered via intraperitoneal (i.p.) injection. After an interval of 46–48 h, hCG (5–10 IU per mouse) was subsequently injected intraperitoneally to trigger ovulation. Oocytes were collected and vitrified 12–14 h after the hCG injection. The cryopreservation and warming of oocytes were performed following the protocol outlined in our previously published study.^32^ Briefly, mouse oocytes were equilibrated at room temperature for 5 min in EM. After equilibration, the oocytes were transferred into vitrification solution, left at room temperature for 1 min and then immediately placed into carrier before being rapidly frozen in liquid nitrogen. 5–10 oocytes were vitrified in each straw (Medsuyun, China). The fresh control group oocytes were not vitrified and were directly subjected to the intracytoplasmic sperm injection (ICSI) procedure.

First, the oocytes were removed from the cryovials and placed in a warming buffer composed of 1.0 M sucrose and DPBS containing 20% (v/v) FBS. The oocytes were left at 30°C for 3 min. Next, the oocytes were sequentially transferred to solutions containing.5,.25 and 0 M sucrose, each with 20% (v/v) FBS in DPBS, and incubated at room temperature for 3 min. Finally, the treated oocytes were placed in pre‐equilibrated human tubal fluid (HTF) and cultured at 37°C in 5% CO_2_, 95% air and 100% humidity for 2 h. Afterward, the oocytes were examined under an inverted microscope. Oocytes with intact membranes, clear zona pellucida and uniform cytoplasm without fragmentation were considered viable and were used for subsequent in vitro fertilisation experiments.

### 2.4 Oocyte ICSI and embryo culture

Due to vitrification‐induced zona hardening,^33^ ICSI was applied to all warmed oocytes to bypass the impaired natural sperm penetration. Male mice were euthanised by cervical dislocation following anaesthesia. The cauda epididymides were promptly excised and placed into pre‐equilibrated HTF medium (Sage, USA). The epididymal tissue was minced using ophthalmic scissors to release sperm, which were then incubated under standard culture conditions (37°C, 5% CO_2_, 95% air and saturated humidity) for 10 min. ICSI was performed using a micromanipulation system equipped with a piezo micropipette‐driving unit. Motile sperm with normal morphology and mature oocytes with clear first polar bodies and good structural integrity were selected for injection. Following ICSI, oocytes were cultured in vitro for 6 h, and the formation of the second polar body and pronuclei was observed to assess fertilisation quality.

Fertilised oocytes were subsequently transferred into G1 Plus medium (Vitrolife, Sweden) and cultured under standard conditions (37°C, 5% CO_2_, 95% air and saturated humidity) until they reached the 8‐cell stage. The embryos were then promptly transferred into pre‐equilibrated G2 Plus medium (Vitrolife, Sweden) and cultured further until the blastocyst stage. Early 1 cells were collected at about 2–4 h post‐ICSI. Late 1 cells were collected at about 10–12 h post‐ICSI. Early 2 cells were collected at about 18–20 h post‐ICSI. Late 2 cells were collected at about 28–32 h post‐ICSI. Morulas were collected at about 68–72 h post‐ICSI. Blastocysts were collected at about 92–96 h post‐ICSI.

### 2.5 Immunofluorescence staining of oocytes and embryos

Blastocysts were collected and fixed in PBS containing 4% paraformaldehyde (PFA) for 30 min at room temperature. Following fixation, samples were permeabilised with PBS containing.5% Triton X‐100 for 20 min at room temperature and subsequently blocked with blocking buffer (PBS containing 5% bovine serum albumin) for 1 h at room temperature. The samples were then incubated with primary antibodies (anti‐CDX2, Biogenex AM392‐5M, 1:50; anti‐Nanog, Cell Signaling Technology 8822S, 1:200) overnight at 4°C. Following three washes, the samples were incubated with fluorescence‐conjugated secondary antibodies (1:500 dilution) for 1 h and Hoechst 33342 (1:1000 dilution) for 15 min at room temperature in the dark. After final washes, the samples were mounted on glass‐bottom confocal dishes. Confocal images were acquired using a Zeiss LSM 980 scanning microscope. Fluorescence images were analysed using Fiji and Imaris.

### 2.6 Embryo collection and single‐cell dissociation

Blastocysts obtained from culture were transferred into DPBS containing.3% HCl and incubated briefly to remove the zona pellucida. The embryos were then washed several times in DPBS supplemented with.5% HSA and transferred into a digestion solution composed of Accutase and.25% Trypsin–EDTA mixed at a 1:1 ratio. Embryos were incubated in this solution for 10–60 min until cell dissociation became apparent, at which point enzymatic digestion was terminated. The digested embryos were subsequently transferred into droplets of DPBS containing.5% HSA. Gentle pipetting was performed using glass capillaries of varying diameters to dissociate the embryos into single cells. Finally, individual cells were transferred into 200 µL tubes containing lysis buffer using mouth‐controlled pipettes.

### 2.7 cDNA preparation and scRNA‐seq

Individual blastomeres were quickly transferred using a mouth‐controlled pipette into lysis buffer containing RNase inhibitor (40 U/µL), 10% Triton X‐100, primers (10 µM), dNTPs (10 mM) and ERCC spike‐ins. The mixture was thoroughly vortexed and briefly centrifuged, followed by cell lysis at 72°C for 90 s. Reverse transcription was then performed by combining the cell lysate with a reverse transcription master mix containing SuperScript II reverse transcriptase (200 U/µL), RNase inhibitor (40 U/µL), 5× SuperScript II first‐strand buffer, dithiothreitol (.1 M), betaine (5 M), MgCl_2_ (1 M) and TSO primers (100 µM).

Following reverse transcription, primer removal, A‐tailing and second‐strand synthesis were performed. Amplification of the resulting cDNA was carried out using a single round of polymerase chain reaction (PCR) with no more than 24 cycles to generate sufficient cDNA for sequencing library construction. The reverse‐transcribed cDNA was preamplified using 2× KAPA HiFi HotStart ReadyMix along with IS and 3′P2 primers. The PCR products were purified using.8× XP magnetic beads and further amplified to obtain the cDNA quantity required for downstream applications.

### 2.8 Real‐time quantitative PCR

Individual blastocysts were subjected to RNA extraction and cDNA preparation using the same reverse transcription reagents and conditions as described in Section 2.7. RT‐qPCR was carried out using SYBR Green Master Mix (Applied Biosystems; A25742)on the QuantStudio 3 Real‐Time PCR System. Each reaction was run in triplicate, and the amplification specificity was verified by melt curve analysis. Expression levels of target genes were normalised to the endogenous control 18S rRNA and quantified using the 2^−ΔΔCt^ method relative to the control group. Primer sequences used in this study are listed in Table S1.

### 2.9 Library construction and sequencing data processing

Amplified cDNA (5–100 ng) was sheared to approximately 200 bp fragments using Covaris S2 (Covaris, USA) and purified with the DNA Clean & Concentrator™‐5 kit. Library construction was then performed using the NEBNext Ultra DNA Library Prep Kit for Illumina. The cDNA libraries were sequenced on an Illumina HiSeq 2000 or 2500 platform using 100 bp paired‐end reads.

All raw scRNA‐seq data were preprocessed to remove Illumina adapter sequences and 3′ poly‐A tails.^34^ The cleaned reads were aligned to the mouse reference genome (mm10) using STAR software (v2.7.0) in two‐pass mode.^35^ Gene‐level read counts were then obtained using the featureCounts tool,^36^ and transcripts per million (TPM) expression matrices were generated based on gene lengths.

### 2.10 Sequencing depth and quality control

RNA‐seq libraries were sequenced using the Illumina sequencing platform. Raw image data generated by the sequencer were converted into sequence data through base calling. Low‐quality cells were removed by applying a quality control filter, retaining only those with more than 3000 detected genes and a mitochondrial gene fraction of less than 10%. Additionally, genes expressed in fewer than five cells were excluded from further analysis. Filtering and normalisation were performed following the standard workflow of the *Seurat* ^37^ package (v4.0.5), resulting in 28 039 high‐confidence expressed genes retained for downstream analyses.

### 2.11 Embryo sex interpretation

To determine embryo sex, we first selected blastocysts with more than three cells for subsequent investigation. The overall expression levels of X‐linked and Y‐linked gene sets were calculated for each individual cell using TPM values. The Y‐linked gene expression levels across all cells within each embryo were then ranked. Embryos with a median Y‐linked gene expression level greater than 25 were classified as male, while those with a median expression ≤25 were classified as female. Additionally, the ratio of overall X chromosome gene expression to total gene expression was assessed across all cells to further validate embryo sex classification. Based on these criteria, a total of 19 male embryos and 24 female embryos were identified.

### 2.12 Generation and analysis of blastocyst cell clusters

The obtained blastocyst scRNA‐seq data were analysed and annotated using the *Seurat* package. First, the log_2_ (TPM+1) expression matrices from both the fresh and cryo groups were loaded into R using the CreateSeuratObject function, and Seurat objects were constructed separately for each dataset. Only genes expressed in at least five cells were retained for analysis. A total of 738 blastocyst cells were included. Based on the expression patterns of known marker genes,^38^, ^39^ 416 cells were identified as TE and 323 as ICM cells (Table S2).

### 2.13 Differential gene expression and functional annotation of blastocyst cell clusters

Only cell types derived from more than five embryos were included in the analysis. The MAST algorithm was used to identify DEGs between groups at the single‐cell level. Genes with a |log_2_(fold change)| > .5 and a *p*adj < .05 were considered significantly differentially expressed. Compared with the fresh group, a total of five DEGs were identified in the ICM of blastocysts from the cryo group, including one upregulated and four downregulated genes (Table S3). In TE cells, nine DEGs were identified, with one upregulated and eight downregulated (Table S4).

Subsequently, cell clusters yielding more than 3 DEGs were subjected to Gene Ontology (GO) enrichment analysis using the *clusterProfiler* ^40^ package (v4.16.0). Pathways or terms with *p*adj < .05 were considered significantly enriched (Table S5).

### 2.14 *Xist* expression level and annotation

The single‐cell gene expression levels from the blastocyst scRNA‐seq data were quantified as log_2_(TPM+1). Embryonic cells with log_2_(TPM+1) values of *Xist* greater than 3 were defined as *Xist*‐high‐expressing cells, while those with values less than 3 were defined as *Xist*‐low‐expressing cells. The *Xist* expression levels for all blastocyst cells were subsequently annotated (Table S2).

### 2.15 Differentiation potential and pseudotime analysis of cell clusters

The CytoTRACE^41^ package (v1.1.0) was used following its standard pipeline to quantify the number of expressed genes and transcriptional diversity of each TE and ICM cell in blastocysts from different treatment groups. This analysis was employed to predict the differentiation state and potential of distinct cell clusters in both the fresh and cryo groups at the blastocyst stage and to determine their developmental trajectories (Table S2). Subsequently, *Monocle* ^42^ package (v2.18.0) was utilised with default settings to perform pseudotime analysis on various blastocyst cell types. After obtaining the corresponding pseudotime expression matrix of the cell clusters, the DEGs identified in TE and ICM cells by the *Seurat* package were used to reorder the cells along pseudotime. Dimensionality reduction was then carried out using the DDRTree method to define the pseudotemporal sequence of the cells.

### 2.16 Cell cycle phase determination of cell clusters in blastocysts

To determine whether cell cycle dysregulation occurs in blastocyst cells of the cryo group compared with the fresh group, we inferred cell cycle phases based on previously reported gene sets associated with G1/S and G2/M transitions.^43^ Normalised TPM values from embryo scRNA‐seq data were imported into the *Seurat* package, and the CellCycleScoring function was applied to calculate phase scores for each cell based on marker gene expression. Each cell was then categorised into one of the three phases: G1, S or G2/M. This approach enabled a comparative analysis of cell cycle–related gene expression patterns between the cryo and fresh groups at the blastocyst stage.

### 2.17 Gene module scoring and correlation analysis between X chromosome expression and cell cycle

To evaluate the overall expression levels of specific biological modules, the AddModuleScore function in the Seurat package was employed. Specifically, curated gene sets comprising X chromosome‐linked genes and known cell cycle regulators were utilised to calculate the X chromosome expression score and cell cycle score for each individual cell. To further investigate the potential relationship between X chromosome expression and cell cycle regulation across different treatment groups (fresh vs. cryo) and cell lineages (TE and ICM), Pearson correlation analysis was performed. The correlation coefficients (𝑟) and statistical significance (*p* values) were calculated for each subgroup.

### 2.18 Intercellular communication analysis among cell clusters in blastocysts

Cell–cell communication within blastocysts was inferred from normalised scRNA‐seq data using the *CellChat* ^44^ package (v1.1.3), and comparisons were made between the fresh and cryo groups. CellChat quantitatively inferred and analysed intercellular signalling networks based on a curated database of known ligand–receptor interactions and their cofactors. Interaction networks among various cell clusters were constructed by identifying differentially expressed signalling genes within each cluster. During the analysis, ‘CellChatDB.mouse’ was specified as the ligand–receptor interaction reference database, and all parameters were set to default unless otherwise noted. To compare the overall number and strength of intercellular communications between the two groups, CellChat objects were merged using the mergeCellChat function.

### 2.19 Statistical analysis

For continuous variables conforming to a normal distribution, comparisons between groups were conducted using Student's *t*‐test. For non‐normally distributed variables, the Mann–Whitney *U* test was applied. Classification and proportion statistics were analysed using Fisher's exact test (unless otherwise specified). All statistical analyses were performed using SPSS software (version 25.0) or R software. A *p* value or *p*adj‐value of less than.05 was considered indicative of a statistically significant difference between groups.

## 3 RESULTS

### 3.1 Early embryo cell clustering and gene expression

**FIGURE 1:**
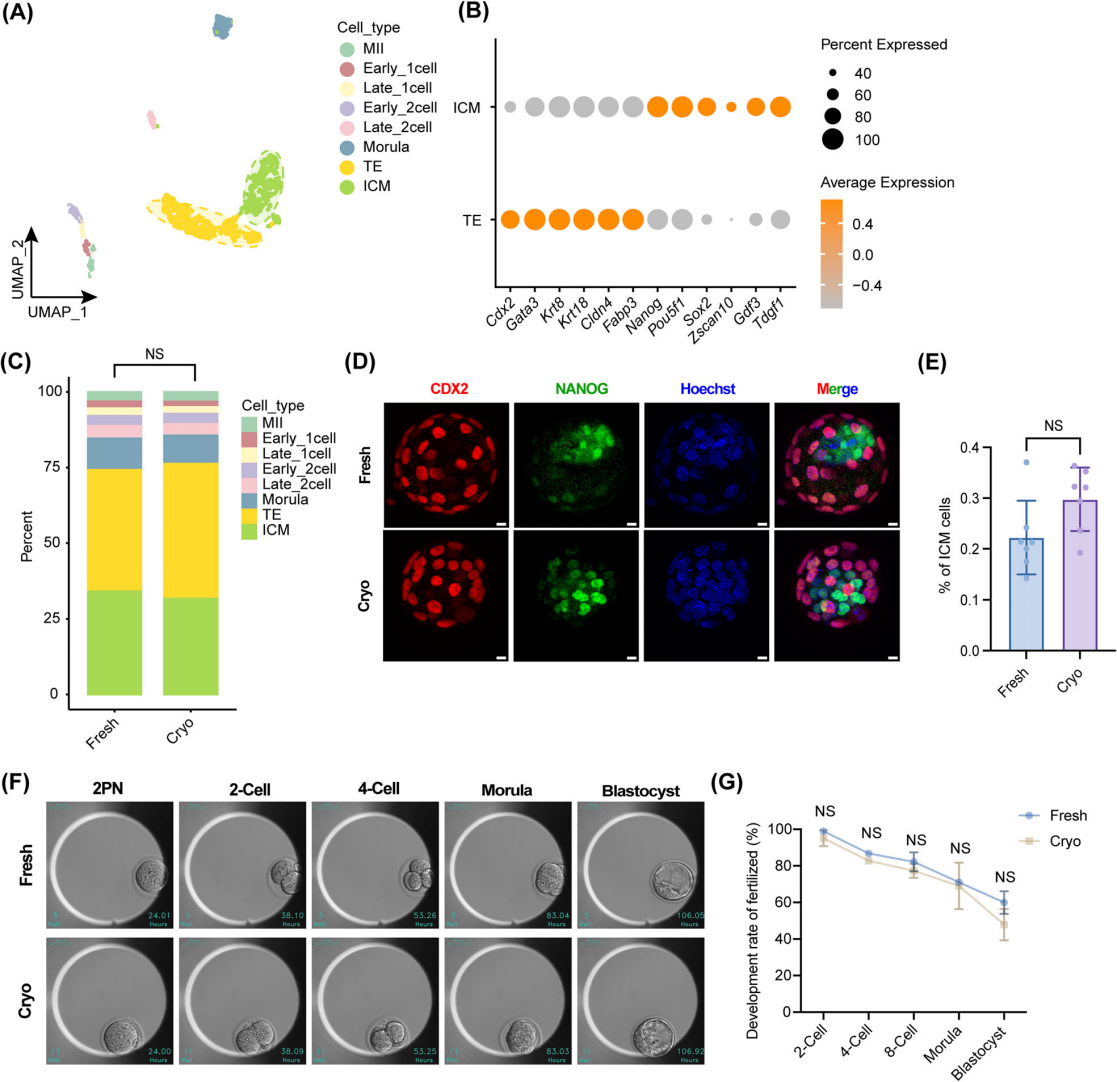
Effects of oocyte cryopreservation on transcriptional profiles of different cell types at the oocyte and early embryo. (A) UMAP plot of the oocyte and early embryo. (B) Expression levels of representative ICM‐ and TE‐specific marker genes in blastocyst cells. Dot size indicates the percentage of cells expressing the gene, while colour intensity from grey to orange reflects increasing expression levels. (C) Proportional composition of oocytes and early embryos across developmental stages in fresh versus cryo groups, NS, *p* > .05. (D) Representative immunofluorescence staining images of blastocysts derived from fresh versus cryopreserved oocytes. The ICM lineage is marked by NANOG (green), and the TE lineage is marked by CDX2 (red). Nuclei were counterstained with Hoechst (blue). Scale bar = 10 µm. (E) Quantification of the proportion of ICM cells relative to total blastocyst cell counts in the fresh (*n* = 11) and cryo (*n* = 8) groups. NS, *p* > .05. (F and G) Representative images and developmental rates of fresh (*n* = 127) and cryo (*n* = 105) groups. Scale bar = 50 µm. NS, *p*  > .05.

The identities of the cell stages, including mature MII oocytes, early/late 1 cell, early/late 2 cell and Morula, were determined at the time of sample collection and were therefore directly annotated during analysis. Unsupervised clustering analysis of all blastocyst cells was performed using the Seurat package, revealing that the cells could be divided into two distinct subclusters, each exhibiting unique gene expression profiles (Figure 1A). Further analysis showed that one cluster exhibited high expression of *Nanog*, *Pou5f1*, *Sox2*, *Zscan10*, *Gdf3* and *Tdgf1*, which are canonical markers of the ICM. Meanwhile, the other cluster showed elevated expression of TE marker genes such as *Cdx2*, *Gata3*, *Krt8*, *Krt18*, *Cldn4* and *Fabp3* (Figure 1B). Based on the expression of these signature genes, blastocyst cells were classified into ICM and TE cluster.

To systematically evaluate the potential effects of oocyte cryopreservation on preimplantation embryonic development, we analysed the cell counts of each cell cluster and found no significant differences in cell numbers across the clusters between the cryo and fresh groups (Figure 1C). Then, we collected oocytes and early embryos from both fresh and cryo groups across various developmental stages for subsequent analyses. At the blastocyst stage, we performed immunofluorescence staining to morphologically validate lineage specification. Blastocysts derived from both fresh and cryopreserved oocytes exhibited clear structural segregation of ICM and TE lineages, with comparable spatial localisation and overall morphology between the two groups (Figure 1D). Based on these immunofluorescence images, we quantified the cellular composition of blastocysts in both groups. Statistical analysis revealed no significant difference in the proportion of ICM cells relative to total blastocyst cell counts between the cryo and fresh groups (Figure 1E). Furthermore, assessment of embryo formation rates confirmed that developmental competence remained uncompromised, with comparable rates observed between the fresh and cryo groups (Figure 1F,G).

To further investigate the impact of oocyte cryopreservation on early embryonic development, DEGs in MII oocytes, early/late 1 cell, early/late 2 cell, ICM and TE clusters were analysed separately. The analysis revealed that oocyte freezing did not cause significant effects in the cell types including MII oocytes, early/late 1 cell and late 2 cell, with no DEGs present in any of the aforementioned cell types (Figure S1A). Only one DEG was identified in the early 2 cell, and a total of five DEGs were identified in the ICM of the cryo group, including one upregulated and four downregulated genes (Figure S1A and Table S3). In the TE cells, nine DEGs were identified, with one upregulated and eight downregulated (Figure S1A and Table S4). These results suggest that oocyte cryopreservation affects a small subset of genes in both ICM and TE cells. Next, we utilised RT‐qPCR to evaluate the expression levels of two DEGs, *Bex1* and *Phlda2*, in intact blastocysts. The results revealed no significant differences in the expression of either *Bex1* or *Phlda2* between the fresh and cryo groups. However, *Bex1* still exhibited a downregulation trend in the cryo group, which is consistent with the scRNA‐seq analysis (Figure S1B).

Furtherly, enrichment analysis was conducted for the five ICM‐specific and nine TE‐specific DEGs in the fresh and cryo groups. GO enrichment analysis revealed differences in the altered biological processes between the TE and ICM lineages. In both the TE and ICM clusters, commonly upregulated genes in the cryo group were enriched in translational elongation, while commonly downregulated genes were enriched in terms such as ectopic germ cell programmed cell death and chromosome organisation involved in meiotic cell cycle. For the TE cluster, downregulated genes were associated with terms such as homologous chromosome pairing at meiosis and homologous chromosome segregation (Figure S1C and Table S5). For the ICM cells, downregulated genes were predominantly enriched in processes including negative regulation of reproductive process and regulation of reproductive process (Figure S1C and Table S5).

### 3.2 Oocyte cryopreservation and embryonic sex interpretation

By analysing the expression levels of specific genes and the relative abundance of X‐ and Y‐linked genes, it was observed that among 43 embryos, 24 exhibited higher expression levels of X chromosome‐specific genes and lower overall expression of Y‐linked genes. The remaining 19 embryos demonstrated elevated expression of Y‐linked genes, while their X‐linked gene expression was relatively lower. Accordingly, 24 embryos were classified as female and 19 as male based on their sex chromosome expression profiles (Table S2 and Figure S2A,B), resulting the overall sex ratio (female to male) of embryos obtained in this study was approximately balanced.

Among the 24 female embryos, 14 originated from the fresh group and 10 from the cryo group. Of the 19 male embryos, 7 belonged to the fresh group and 12 to the cryo group. However, no statistically significant differences in sex ratios were observed between the groups (Figure S2C, *p* > .05).

### 3.3 Developmental dynamics and sex‐specific *Xist* regulation in early embryos

**FIGURE 2:**
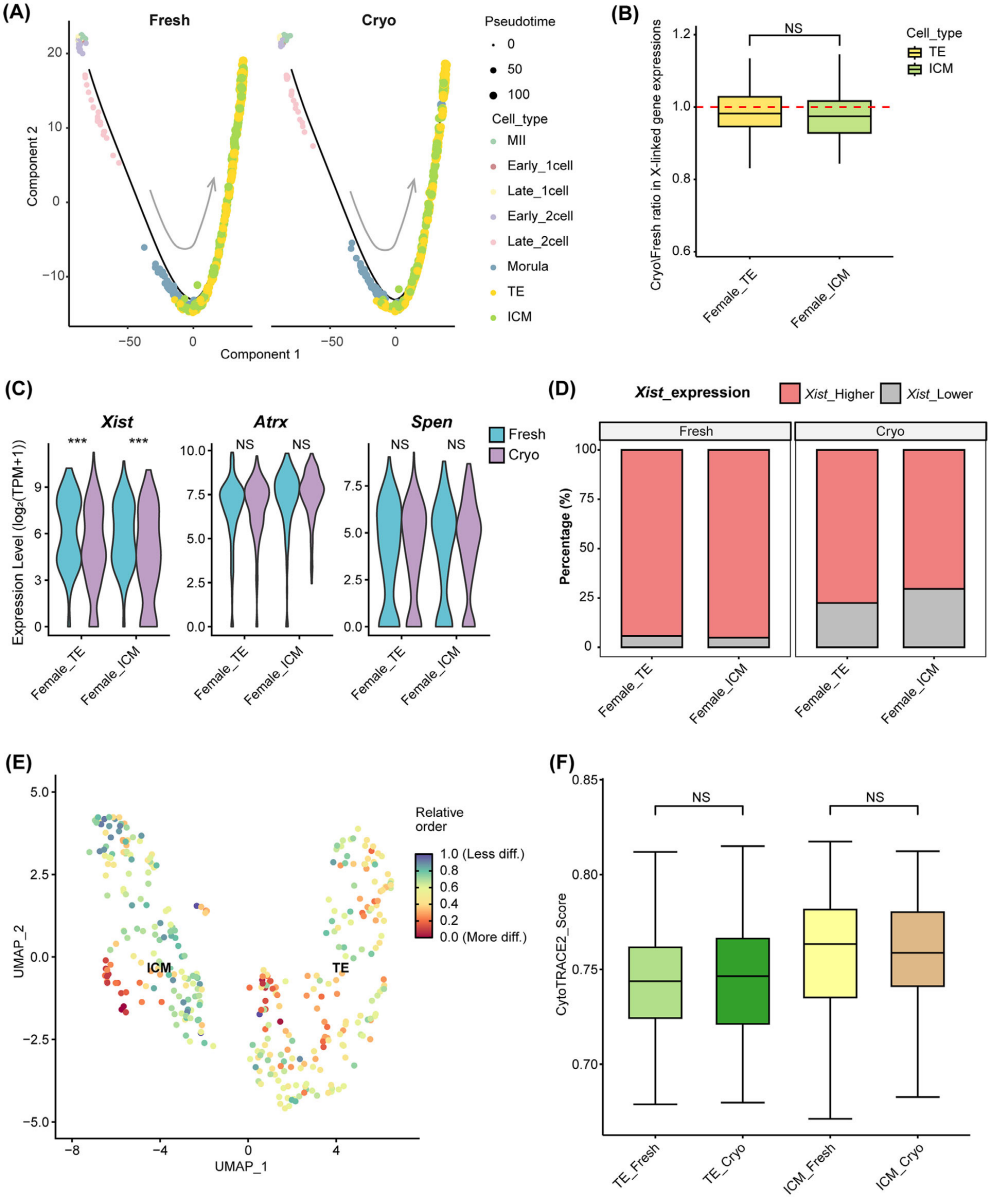
Effects of oocyte cryopreservation on developmental trajectories and differentiation of blastocyst cell clusters. (A) The pseudotime plot illustrates the developmental trajectory from MII oocytes to cells at various blastocyst stages, with the fresh group shown on the left and the cryo group on the right. Different colours represent distinct cell clusters, and the progression from small to large dots indicates the developmental direction from early to late stages. (B) Relative expression levels of X‐linked genes in female TE and ICM cells from the cryo group compared with the fresh group. The red dashed line indicates a ratio of 1.0; NS, *p *> .05. (C) Violin plots showing the expression levels of *Xist*, *Atrx* and *Spen* in TE and ICM in female cells of blastocysts. The *y*‐axis represents log_2_ (TPM+1) values; NS, *p* > .05; ^***^, *p* < .001. (D) Bar plots showing the proportions of cells with high and low *Xist* expression in TE and ICM cell populations of female blastocysts from the cryo and fresh groups. (E) UMAP plot of the overall differentiation potential of ICM and TE cell clusters in fresh group. Dot colours range from blue to red, indicating increasing relative pluripotency; lower numerical values correspond to higher differentiation potential. (F) Boxplots of differentiation scores for ICM and TE cell populations in blastocysts derived from fresh and cryo oocytes. The y‐axis represents the differentiation score. NS, *p* > .05.

Trajectory analysis of MII oocytes and early embryonic cells at various stages revealed that cells from each stage in both the fresh and cryo groups exhibited a progressive transition from a naïve state to a more differentiated state, with highly comparable developmental trajectories observed between the two groups (Figure 2A). These findings indicate that oocyte cryopreservation does not substantially alter the direction of differentiation or the developmental progression from oocytes to blastocysts. To further determine whether oocyte cryopreservation affects the stability of X‐linked gene expression in blastocyst cells, we compared the proportion of reads mapped to X‐linked genes relative to total gene expression between the fresh and cryo groups. In female TE cells, the ratio of X‐linked gene expression in the cryo group relative to the fresh group was approximately .980; in female ICM cells, this ratio was approximately .977 (Figure 2B); similarly, in male TE cells, the ratio of X‐linked gene expression in the cryo group relative to the fresh group was approximately .987 and in male ICM cells, this ratio was approximately .993 (Figure S3A). Both values are close to 1, indicating that oocyte cryopreservation does not significantly alter the overall expression level of X‐linked genes in blastocyst cells, thereby maintaining the normal expression dosage of the X chromosome in both female and male cells without adversely affecting embryonic development (Figures 2B and S3A).

At a specific stage of normal female blastocyst development, massive expression of *Xist* on one of the X chromosomes is required for survival and proper development, thereby achieving dosage compensation. Unlike females, in normal male blastocysts, *Xist* must be maintained at minimal basal levels to prevent the lethal silencing of the single X chromosome. Interestingly, our single‐cell analysis revealed that in both female and male blastocysts, the fresh group exhibited slightly higher *Xist* expression levels, but this did not alter the expression of *Atrx* and *Spen* (Figures 2C and S3B). Following oocyte cryopreservation, female blastocysts showed a higher proportion of *Xist* low‐expressing cells (Figure 2D), whereas male blastocysts exhibited a higher proportion of *Xist* low‐expressing cells exclusively in the ICM (Figure S3C). Separate statistical analyses of cells with different *Xist* expression profiles in female and male TE and ICM clusters after cryopreservation revealed that the proportion of *Xist* low‐expressing cells was significantly increased in both female TE and ICM clusters of the cryo group (Table 1; *p* < .001). Furthermore, the male cryo group also showed a significant increase in the proportion of *Xist* low‐expressing cells in the ICM cluster (Table 1; *p* < .05). Subsequently, we mapped the *Xist* high‐expressing cells (marked in red) from the fresh and cryo groups onto the embryonic developmental trajectory, with cells of both sexes highlighted respectively. We found that on the pseudotime plot, the fresh group exhibited a greater number of *Xist* high‐expressing cells in both female (Figure S3D) and male embryos (Figure S3E) compared with the cryo group.

**TABLE 1:** The proportion of different *Xist*‐expressing cells.

Sex	Cell_type	Group	*Xist*_expression	Cell_Num (%)	*p*
Female	TE	Fresh	*Xist*_Higher	182 (94.30%)	*p* < .05
			*Xist*_Lower	11 (5.70%)	
		Cryo	*Xist*_Higher	173 (77.58%)	
			*Xist*_Lower	50 (22.42%)	
	ICM	Fresh	*Xist*_Higher	156 (95.12%)	*p* < .05
			*Xist*_Lower	8 (4.88%)	
		Cryo	*Xist*_Higher	112 (70.44%)	
			*Xist*_Lower	47 (29.56%)	
Male	TE	Fresh	*Xist*_Higher	15 (25.00%)	*p* > .05
			*Xist*_Lower	45 (75.00%)	
		Cryo	*Xist*_Higher	28 (21.37%)	
			*Xist*_Lower	103 (78.63%)	
	ICM	Fresh	*Xist*_Higher	20 (29.85%)	*p* < .05
			*Xist*_Lower	47 (70.15%)	
		Cryo	*Xist*_Higher	11 (12.79%)	
			*Xist*_Lower	75 (87.21%)	

To further assess differentiation potential, we employed the CytoTRACE package to quantify the developmental potency of all fresh group blastocyst cells (Figure 2E). Further comparative analysis demonstrated that oocyte cryopreservation maintained this developmental capacity in both cell types, with no significant alteration observed between fresh and cryo groups. Statistical analysis confirmed the absence of significant differences between experimental groups (Figure 2F).

### 3.4 Effects of oocyte cryopreservation on cell cycle regulation in different blastocyst cell types

**FIGURE 3:**
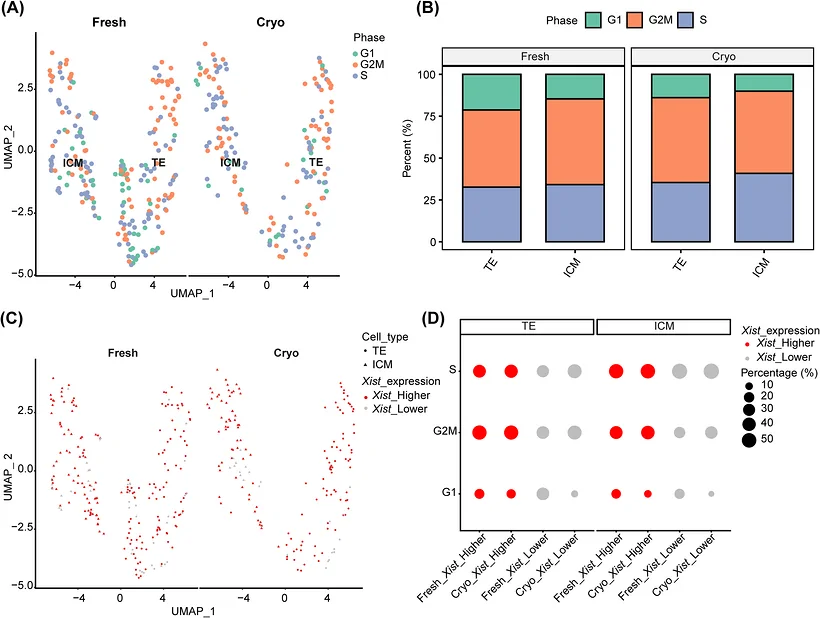
Effects of oocyte cryopreservation on cell cycle in female blastocyst cells. (A) Dimensionality reduction plots showing the assignment of cell cycle phases (G1, S and G2/M) across all cells from MII oocytes to the blastocyst stage. Cells are coloured by cell cycle phase and split by the fresh (left) and cryo (right) groups. (B) Bar plots illustrating the relative proportions of cells in the G1, S and G2/M phases across distinct developmental clusters in the fresh and cryo groups. (C) Dimensionality reduction plots highlighting the distribution of *Xist*‐higher expressing cells (marked in red) specifically within female blastocysts. Cell types are indicated by distinct shapes (circles for TE, triangles for ICM), and the plots are split by the fresh (left) and cryo (right) groups. (D) Dot plots illustrating the cell cycle phase distribution patterns of *Xist*‐higher (red) and *Xist*‐lower (grey) cells within the TE and ICM subpopulations of female blastocysts. The size of the dots represents the percentage of cells at the specified cell cycle stage.

During early embryonic development in mammals, mitotic activity remains high, and the expression of genes associated with cell cycle regulation is essential for maintaining proper cell division and differentiation. To investigate whether oocyte cryopreservation affects cell cycle regulation in cells of subsequent blastocysts, we analysed cell cycle‐related gene expression within distinct blastocyst cell clusters using the *Seurat* package. To begin with, cell cycle phase scoring and clustering analysis were performed for all blastocyst cells in both the fresh and cryo groups (Figure 3A). Blastocyst cells from both the fresh and cryo groups were distributed across the G1, G2/M and S phases (Figure 3B). Then, we quantified the number and proportion of cells in different cell cycle phases across each cell cluster in the fresh and cryo groups. Compared with the fresh group, the cell cycle proportions in the TE and ICM cell clusters of the cryo group showed no significant changes (Table 2; *p* > .05).

**TABLE 2:** The proportion of cells in different cell cycles.

Cell_type	Group	Phase	Cell_Num (%)	*p*
TE	Fresh	G1	41 (21.24%)	*p* > .05
		G2M	89 (46.11%)	
		S	63 (32.64%)	
	Cryo	G1	31 (13.90%)	
		G2M	113 (50.67%)	
		S	79 (35.43%)	
ICM	Fresh	G1	24 (14.63%)	*p* > .05
		G2M	84 (51.22%)	
		S	56 (34.15%)	
	Cryo	G1	16 (10.06%)	
		G2M	78 (49.06%)	
		S	65 (40.88%)	

Subsequently, the expression patterns of four key cell cycle–regulating genes, such as *Pcna*, *Top2a*, *Mcm6* and *Mki67*, in all embryonic cells from both groups were analysed. The expression levels and distribution of these genes were similar between the fresh and cryo groups (Figure S4A), suggesting that oocyte cryopreservation did not significantly alter cell cycle related gene expression at the blastocyst stage. To visualise the cell cycle progression, we performed dimensionality reduction analysis on female embryonic cells, colouring each cell by its assigned cell cycle phase (G1, S or G2/M) and faceting by group and cell type (Figure S4B). In both the fresh and cryo groups, cells from the ICM and TE were distributed across all phases of the cell cycle. Notably, no obvious clustering of any particular phase was observed exclusively in the cryo group, suggesting that cryopreservation did not grossly alter the cell cycle dynamics of pre‐implantation embryos.

Next, we highlighted *Xist*‐higher expressing cells in both female and male blastocysts in red and found that a large number of these *Xist*‐higher expressing cells were also scattered within both the TE and ICM subpopulations (Figures 3C and S4C). In female blastocysts, the fresh and cryo groups exhibited similar proportions of *Xist*‐higher and *Xist*‐lower cells in the S and G2/M phases within both the TE and ICM, whereas the fresh group showed a higher proportion of cells in the G1 phase (Figure 3D). Regarding male cells, the proportion of *Xist*‐higher cells in the ICM of the cryopreservation group was higher in the S and G1 phases, whereas the fresh group exhibited a higher proportion of these cells in the G2/M phase of ICM cells (Figure S4D). Subsequently, statistical analysis of the number and proportion of *Xist*‐higher expressing cells across different cell cycle stages within the TE and ICM clusters of both female and male blastocysts revealed that oocyte cryopreservation did not cause a significant difference in the proportion of *Xist*‐higher expressing cells in either the TE or ICM clusters of both female (Table 3; *p* > .05) and male (Table S6; *p* > .05) blastocysts.

**TABLE 3:** The proportion of different *Xist*‐expressing cells in female across different cell cycles.

Cell_type	Group	*Xist*_expression	Phase	Cell_Num (%)	*p*
TE	Fresh	*Xist*_Higher	G1	18 (17.48%)	*p *> .05
		*Xist*_Higher	G2M	49 (47.57%)	
		*Xist*_Higher	S	36 (34.95%)	
		*Xist*_Lower	G1	10 (34.48%)	
		*Xist*_Lower	G2M	10 (34.48%)	
		*Xist*_Lower	S	9 (31.03%)	
	Cryo	*Xist*_Higher	G1	12 (14.81%)	
		*Xist*_Higher	G2M	38 (46.91%)	
		*Xist*_Higher	S	31 (38.27%)	
		*Xist*_Lower	G1	1 (9.09%)	
		*Xist*_Lower	G2M	5 (45.45%)	
		*Xist*_Lower	S	5 (45.45%)	
ICM	Fresh	*Xist*_Higher	G1	13 (16.05%)	*p* > .05
		*Xist*_Higher	G2M	29 (35.80%)	
		*Xist*_Higher	S	39 (48.15%)	
		*Xist*_Lower	G1	3 (18.75%)	
		*Xist*_Lower	G2M	4 (25.00%)	
		*Xist*_Lower	S	9 (56.25%)	
	Cryo	*Xist*_Higher	G1	6 (9.84%)	
		*Xist*_Higher	G2M	25 (40.98%)	
		*Xist*_Higher	S	30 (49.18%)	
		*Xist*_Lower	G1	1 (8.33%)	
		*Xist*_Lower	G2M	4 (33.33%)	
		*Xist*_Lower	S	7 (58.33%)	

### 3.5 Correlation between X‐linked gene expression and cell cycle dynamics during preimplantation development

**FIGURE 4:**
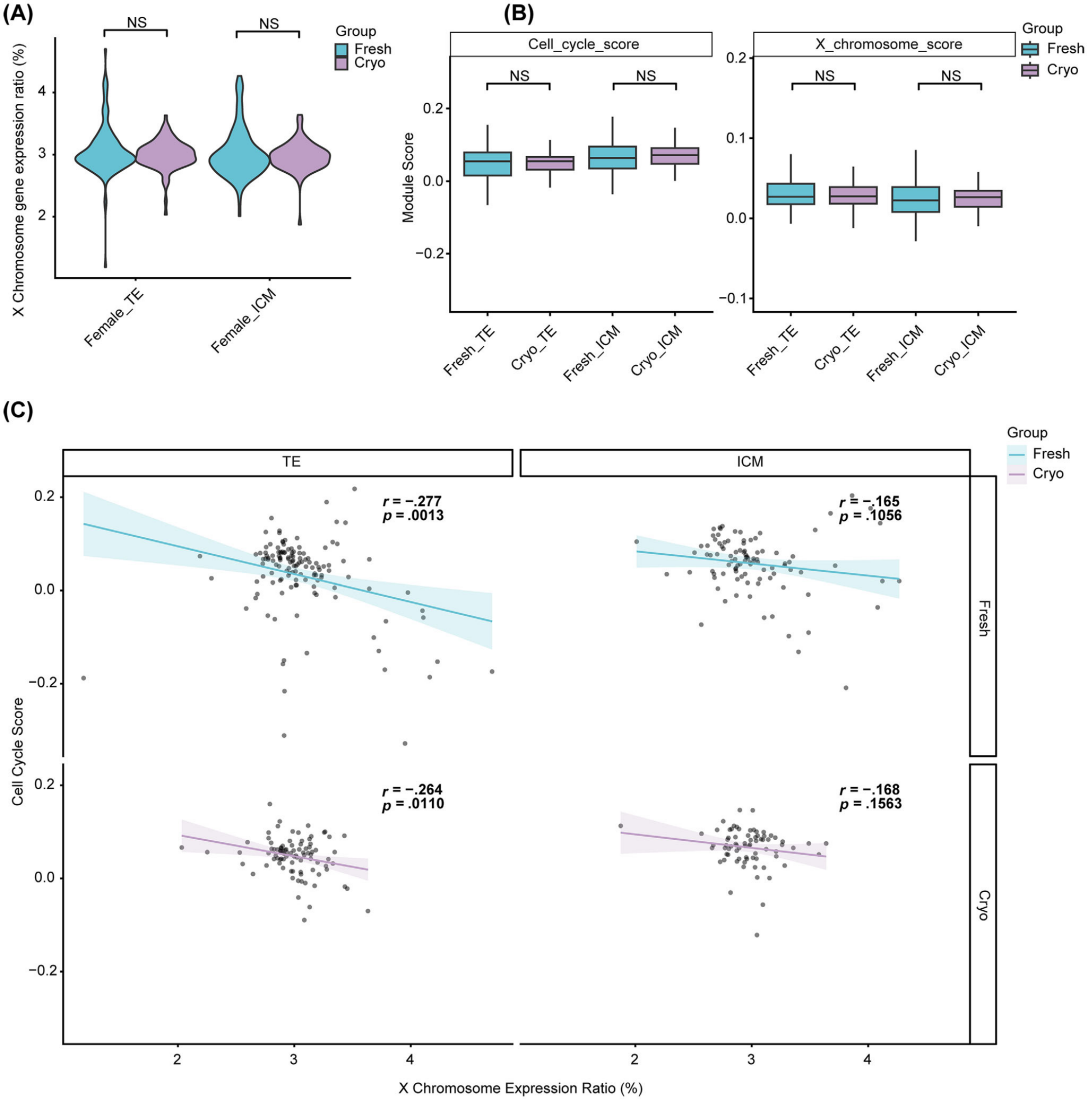
Correlation between X‐linked gene expression and cell cycle dynamics during preimplantation development. (A) The relative proportion of X‐linked gene expression across blastocyst stages in the fresh and cryo groups. NS, *p* > .05. (B) Distribution of the calculated module scores for cell cycle and X‐chromosome gene expression across all identified cell clusters in fresh and cryo groups. (C) Pearson correlation analysis comparing the cell cycle and X‐chromosome module scores within each cell cluster.

To investigate the potential relationship between X‐linked gene expression and cell cycle regulation following oocyte cryopreservation, we evaluated the relative expression levels of X‐linked genes across all cell populations of female blastocysts. Overall, the proportion of X‐linked gene expression remained at approximately 3% across all female blastocyst cell populations. Furthermore, compared with the fresh group, the cryo group exhibited no significant differences in the relative expression levels of X‐linked genes in either the TE or ICM clusters (Figure 4A), establishing a foundation to explore their global correlation with the cell cycle. Subsequently, we calculated the module scores for both the cell cycle and X‐chromosome expression across all female embryonic cell populations. In both groups, the cell cycle module scores were at a relatively low level at the blastocyst stage; moreover, compared with the fresh group, the cell cycle scores of TE and ICM cells in the cryo group showed no significant differences (Figure 4B). Similarly, the X‐chromosome module scores maintained a constantly low level across all cell types irrespective of cryopreservation, and no significant differences in X‐chromosome module scores were observed in the TE and ICM clusters of the cryo group compared with the fresh group (Figure 4B). Furthermore, clustering analysis confirmed that the X‐chromosome and cell cycle scores did not exhibit distinct distribution patterns between the fresh and cryo groups (Figure S4E).

We next sought to determine whether correlations exist between cell cycle dynamics and X‐linked gene expression. By comparing the similarity of the two module scores, we identified a stage‐specific negative correlation between the cell cycle and X‐chromosome gene expression in the TE and ICM cell clusters at the blastocyst stage of both fresh and cryo groups, with Pearson correlation coefficients of −.277 (fresh) and −.264 (cryo) in the TE clusters, and −.165 (fresh) and −.168 (cryo) in the ICM clusters (Figure 4C). Notably, while the correlation coefficients were relatively low, a statistically significant but weak negative correlation was observed in the TE clusters for both the fresh and cryo groups (Figure 4C). However, no significant correlation was detected in the ICM clusters (Figure 4C). Collectively, these findings suggest a weak coordination between X‐chromosome activity and cell cycle progression at the blastocyst stage, whereas the ICM cells, which form the main body of the foetus, remain largely unperturbed by oocyte cryopreservation.

### 3.6 Effects of oocyte cryopreservation on interactions between ICM and TE cells at blastocyst stage

**FIGURE 5:**
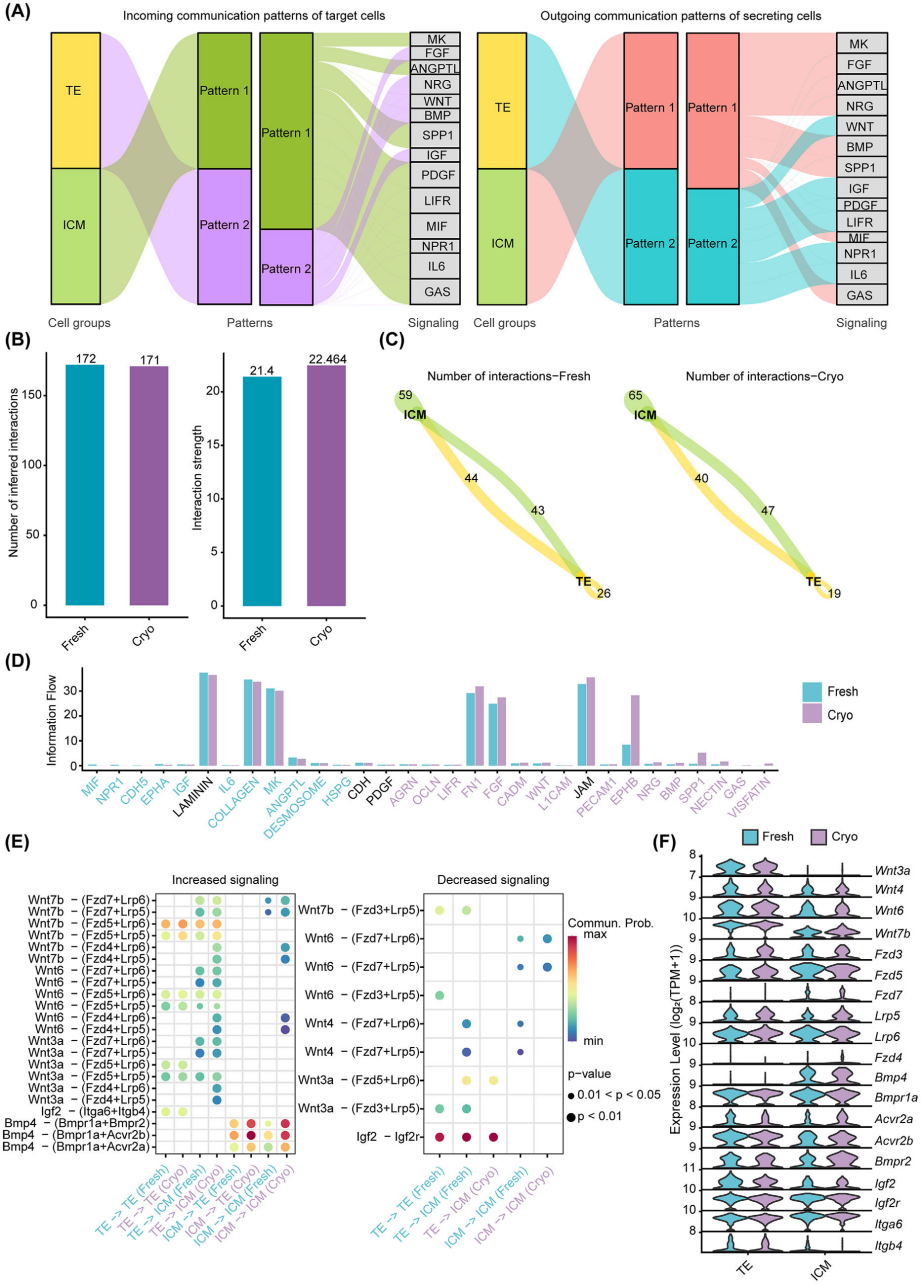
Intercellular communication networks between TE and ICM in blastocysts following oocyte cryopreservation. (A) The inferred outgoing (left) and incoming (right) signalling patterns among TE and ICM cells. The flows map the correspondence between cell groups (left), latent patterns identified by non‐negative matrix factorisation (middle) and specific signalling pathways (right). (B) Bar plots comparing the global number of interactions and overall interaction strength within the blastocyst cells between the fresh and cryo groups. (C) The exact number of autocrine and paracrine interactions among the ICM and TE populations. Edge thickness represents the frequency of interactions in the fresh (left) and cryo (right) groups. (D) Comparing the overall information flow (signalling activity) of representative pathways between the two groups. (E) The predicted communication probabilities of specific ligand–receptor pairs within the WNT, BMP and IGF signalling networks. The plots highlight ligand–receptor pairs that exhibit increased or decreased signalling in the cryo group compared with the fresh group. (F) The relative expression levels of key ligand and receptor genes involved in the selected pathways (WNT, BMP and IGF) across the datasets.

To systematically evaluate whether oocyte cryopreservation and subsequent warming alter intercellular signalling networks within blastocyst cells, the CellChat package was employed to infer autocrine and paracrine signalling activities between TE and ICM populations. CellChat characterises intercellular communication based on a curated database of ligand–receptor interactions, using scRNA‐seq data as input. Through non‐negative matrix factorisation, 14 major signalling pathways involving ICM and TE cells were consolidated into two distinct patterns, representing the predominant cellular contributors to ligand secretion (‘outgoing signalling’) and receptor expression (‘incoming signalling’), respectively. All ICM and TE cells from the blastocysts were mapped to a unique signalling pattern (Figure 5A). The pathway‐specific distributions associated with each pattern were consistent with known embryonic developmental signalling networks.

Following the establishment of these signalling patterns, we compared the overall communication networks between the two groups. Notably, oocyte cryopreservation did not lead to apparent alterations in either the number or the interaction strength of intercellular communications within the blastocyst (Figure 5B). Quantitative analysis of the interaction counts revealed that both autocrine and paracrine signalling events exhibited only minor changes in the cryo group. Specifically, autocrine interactions increased from 59 to 65 within the ICM, whereas they decreased from 26 to 19 within the TE. Correspondingly, reciprocal paracrine interactions also underwent slight shifts in the cryo group, with ICM‐to‐TE signalling decreasing from 44 to 40, and TE‐to‐ICM signalling increasing from 43 to 47 (Figure 5C).

To identify the specific signalling pathways driving these network alterations, we assessed the overall information flow of the interactive pathways. Ranking analysis revealed distinct pathway activation preferences: critical embryo‐development‐related pathways, including the BMP and WNT signalling networks, exhibited significantly higher activity in blastocysts derived from the cryo group. Conversely, the IGF signalling pathway was predominantly more active in the fresh group (Figure 5D).

We next performed a detailed ligand–receptor pair analysis to pinpoint the precise molecular mediators underlying these pathway‐level differences. The results demonstrated that the heightened WNT signalling in the cryo group was primarily driven by enhanced autocrine communications within the TE (TE‐to‐TE) and paracrine signalling from TE to ICM. Meanwhile, the elevated BMP signalling (particularly Bmp4‐mediated interactions) in the cryo group was mainly attributed to increased paracrine crosstalk from ICM to TE, as well as enhanced autocrine signalling within the ICM (ICM‐to‐ICM). In contrast, while WNT signals increased, specific WNT ligand–receptor pairs (such as those involving Wnt4 and Wnt6) showed decreased communication probabilities during both TE autocrine signalling and heterotypic interactions from TE to ICM in the cryo group. Consistent with the pathway‐level downregulation, IGF‐mediated ligand–receptor interactions (specifically the Igf2–Igf2r pair) were dramatically attenuated in TE autocrine signalling, as well as in the paracrine crosstalk from TE to ICM following cryopreservation (Figure 5E).

Finally, to substantiate these inferred communication dynamics at the transcriptional level, we profiled the expression of key genes encoding the ligands and receptors within the WNT, BMP and IGF pathways. The differential expression patterns of these key genes closely mirrored the observed alterations in interaction probabilities across datasets (Figure 5F), further confirming that oocyte cryopreservation subtly exerts a minor influence on certain key developmental signalling networks in the blastocyst.

## 4 DISCUSSION

Although the clinical application of oocyte cryopreservation has steadily increased in recent years, the safety and efficacy of this cryopreservation technique remain subjects of ongoing debate. However, when evaluating the safety and effectiveness of oocyte cryopreservation, individual variability must be taken into account. While the effects of cryopreservation on oocytes have been reported, previous studies concerning its impact on subsequent embryonic development have primarily focused on a few key genes.^19^, ^26^ Consequently, the global transcriptomic landscape of embryos derived from vitrified oocytes remains poorly understood. Moreover, technical constraints previously precluded the cell‐type‐specific analysis of blastocysts derived from cryopreserved oocytes.

In this study, we employed scRNA‐seq to systematically evaluate the impact of oocyte cryopreservation on oocytes and early embryonic development among early/late 1 cell, early/late 2 cell, morula, TE and ICM. This study provides important insights into the molecular mechanisms by which oocyte cryopreservation may elicit subtle influences on embryo. Our results demonstrated that just one DEG was found from the MII stage to the late 2 cell stage between the fresh and cryo groups, suggesting that cryopreservation does not induce major transcriptional perturbations during early cleavage (Figure S1A). Although a small subset of DEGs was identified in the ICM and TE of blastocysts, their limited number underscores the resilience of the embryonic program. The minimal transcriptomic changes in blastocyst lineages (ICM and TE) following oocyte cryopreservation corroborating its overall safety in ART. However, the identified DEGs precisely match established ART stress biomarkers. Specifically, dysregulated imprinted gene *Phlda2* aligns with known cryo‐induced expression fluctuations.^45^ Similarly, altered adhesion molecules like *Cdh2* mirror the reported E‐cadherin reduction under cryo‐stress.^46^ These consistencies strongly validate the biological reliability of our transcriptomic findings. Although scRNA‐seq identified subtle transcriptomic alterations in specific cell lineages (*Bex1* in the ICM and *Phlda2* in the TE), whole‐blastocyst RT‐qPCR revealed no significant overall differences between the fresh and cryo groups (Figure S1B). The absence of significant systemic differences at the whole‐embryo level suggests that these lineage‐specific transcriptomic fluctuations are minor and do not compromise the global developmental program, further underscoring the transcriptomic stability and overall safety of oocyte cryopreservation.

Furthermore, the highly consistent global developmental trajectories (Figure 2A) and the strictly conserved differentiation potential of the ICM (Figure 2F) robustly demonstrate that the core developmental program of the blastocyst remains intact. Although previous studies reported that oocyte cryopreservation can induce a temporary deceleration or delayed timing in subsequent preimplantation embryonic development,^47^, ^48^, ^49^ this lineage‐specific developmental delay appears to be an adaptive fine‐tuning of differentiation dynamics, rather than fundamentally compromising the global developmental integrity of the embryo. Previous studies have reported that epigenetic regulation, including the expression of long non‐coding RNAs and the process of XCI, might be affected following cryopreservation.^50^ In this study, to assess whether oocyte cryopreservation influences the stability of X‐linked gene expression in both female and male blastocysts, we analysed and compared the relative abundance of X‐linked gene reads between the fresh and cryo groups. The values in TE and ICM were both close to 1 for both sexes, suggesting that the overall X‐linked gene expression in male and female blastocyst cells remains comparable following oocyte cryopreservation (Figures 2B and S3A). Although the expression levels of key XCI regulatory genes, *Atrx* and *Spen*, were not significantly changed in the whole cryo group, *Xist* was downregulated in the TE and ICM of both female and male blastocysts (Figures 2C and S3B). Regarding the alterations in *Xist* expression, our sex‐stratified analysis revealed an intriguing sex‐specific transcriptional response to cryopreservation. While male blastocysts showed only minor fluctuations, the downregulation of *Xist* was substantially more pronounced in female blastocysts of the cryo group. Biologically, *Xist* is well characterised as the master non‐coding RNA involved in XCI during normal female embryonic development.^15^ As preimplantation embryos undergo dynamic molecular reprogramming, they are known to be highly sensitive to environmental stressors, such as cryopreservation.^51^ Studies have demonstrated that cryopreservation can induce subtle molecular perturbations in early embryos.^52^, ^53^ In our dataset, the prominent dysregulation of *Xist* specifically in female embryos suggests that the transcriptional networks governing early developmental events may respond to cryo‐stress in a sex‐dependent manner. Nonetheless, from the broader perspective of developmental safety in ART, these sex‐differential transcriptional shifts indicate that male and female embryos may exhibit distinct cryotolerance.

Fertilisation reactivates the cell cycle, allowing mitotic divisions to proceed, and accurate progression of the cell cycle is critical for early embryonic development.^54^ Previous studies have reported that cryopreservation of mouse zygotes and 8‐cell embryos may lead to disruptions in gene expression profiles and perturbations in cell cycle regulation.^55^ However, the impact of oocyte cryopreservation on cell cycle progression during later stages of embryonic development has been less extensively investigated. Our study observed that after oocyte cryopreservation, in each cell cluster during development to blastocysts, the cell cycle proportions in the cryo group showed no significant changes compared with that in fresh group (Table 2).

During the transition to ZGA at the 2‐cell stage, mouse embryos exhibit a marked cell cycle deceleration, primarily through G2 phase elongation. This shift is believed to facilitate the recruitment of transcriptional complex by providing an extended temporal window.^56^ Upon entering the blastocyst stage, the initiation of cellular differentiation coincides with a decoupling of the global cell cycle‐transcription nexus. This regulatory transition is characterised by the emergence of lineage‐specific epigenetic controls—such as imprinted or random XCI—which take over the governance of X‐linked gene expression. Consequently, the transcriptional output of the X chromosome is no longer predominantly driven by cell cycle kinetics, but is instead dictated by localised chromatin states and epigenetic signatures. The progression rate of the cell cycle is highly coordinated with the global wave of ZGA, which encompasses the rapid upregulation of X‐linked transcripts. We found that in cryopreserved oocytes developed to the blastocyst stage, only a weak negative correlation existed between cell cycle module scores and X‐linked gene expression in TE and ICM cell populations (Figure 4C), which was consistent with previous findings. The consistent cell cycle phase distributions and the remarkably constant X‐chromosome module scores across groups in blastocyst cell populations suggest that cryopreserved oocytes maintain the physiological rhythm of cell division and the balance of dosage compensation upon fertilisation.

Intercellular signalling has been considered a crucial aspect in maintaining cellular homeostasis during embryonic development, and imbalances in signalling pathways may contribute to developmental abnormalities. Studies have suggested that transcriptomic features and gene expressions might be highly sensitive to stress responses induced by oocyte cryopreservation.^26^, ^57^ Cell–cell communication analysis revealed no significant increase in either the number or strength of intercellular communications between TE and ICM populations in the cryo group. Although minor changes happened in the cryo group, particularly driven by the signalling pathways like BMP and WNT, etc. (Figure 5), the overall robustness of the communication network appears to successfully support the functional integrity of the blastocyst.

In this study, scRNA‐seq was conducted on cryopreserved MII oocytes as well as early/late 1 cell, early/late 2 cell, morula, and blastocyst‐stage embryos derived from thawed oocytes. The single‐cell transcriptomic profiling reveals that oocyte cryopreservation largely preserves global transcriptomic stability and lineage differentiation in blastocysts. We demonstrated that only a limited subset of genes showed expression alterations at the blastocyst stage after oocytes cryopreservation. However, since this study is confined to the mouse early cleavage and blastocyst stage, the necessity for further exploration of post‐transplantation development and adult health as well as other stress‐control groups remains, as these subtle molecular modifications may have potential long‐term impacts. Consequently, our findings underscore the need for more stringent clinical indications and thorough patient counselling concerning associated risks.

## AUTHOR CONTRIBUTIONS

Liying Yan, Qilong He, Zhiqiang Yan and Yixin Ren obtained funding. Yixin Ren and Qingyang Zhang performed most experiments with the help of Fanqing Xu, Tianyi Liao, Xiaohui Zhu, Nan Wang and Mengyuan Zhao. Qilong He, Qingyang Zhang, Peng Yuan, Zhiqiang Yan and Yao Li performed data analysis. Yixin Ren, Qilong He and Qingyang Zhang wrote the manuscript and revised the article. Qilong He, Liying Yan, Jie Yan, Jie Qiao and Gary D Smith supervised the research. All authors read and approved the final manuscript.

## CONFLICT OF INTEREST STATEMENT

The authors declare no conflicts of interest.

## ETHICS STATEMENT

The experiment was conducted in strict accordance with the guidelines set by the Ethics Committee of Peking University Third Hospital (ethics approval number: A2021315).

## CONSENT FOR PUBLICATION

All the author agreed to publish this paper.

## Supporting information



Supporting Information

Supporting Information

Supporting Information

Supporting Information

Supporting Information

## Data Availability

All sequencing data have been deposited in the Genome Sequence Archive in Beijing Institute of Genomics (BIG) Data Center, Chinese Academy of Sciences under accession codes CRA031734 that are publicly accessible at https://ngdc.cncb.ac.cn/gsa/.
