# Supplementary material for: Single‐cell transcriptomic analysis reveals subtle fluctuations of oocyte cryopreservation on mouse early embryo development
Source: Clin Transl Med. 2026 Sep 7;16(9):e70801. doi: 10.1002/ctm2.70801 (PMC13550515; doi:10.1002/ctm2.70801)
Supplement: Supplementary file 1 [file CTM2-16-e70801-s002.docx]

Supplementary Materials for

**Single-Cell Transcriptomic Analysis Reveals Subtle Fluctuations of Oocyte Cryopreservation on Mouse Early Embryo Development**

**Author:**

Yixin Ren^1,2,3,4,5#^, Qingyang Zhang^1,2,3,4,5#^, Fanqing Xu^1,2,3,4,5^, Yao Li^1,2,3,4,5^, Tianyi Liao^4,5,6^, Mengyuan Zhao^7^, Zhiqiang Yan^1,2,3,4,5^, Xiaohui Zhu^1,2,3,4,5^, Nan Wang^1,2,3,4,5^, Peng Yuan^1,2,3,4,5^, Gary D Smith^8*^, Jie Qiao^1,2,3,4,5*^, Jie Yan^1,2,3,4,5*^, Liying Yan^1,2,3,4,5*^, Qilong He^1,2,3,4,5,9*^

**Affiliations:**

1. Center for Reproductive Medicine, Department of Obstetrics and Gynecology, Peking University Third Hospital, Beijing, China

2. State Key Laboratory of Female Fertility Promotion, Center for Reproductive Medicine, Peking University Third Hospital, Beijing, China.

3. National Clinical Research Center for Obstetrics and Gynecology, Peking University Third Hospital, Beijing, China.

4. Key Laboratory of Assisted Reproduction, Peking University, Ministry of Education, Beijing, China.

5. Beijing Key Laboratory of Reproductive Endocrinology and Assisted Reproductive Technology, Beijing, China.

6. Peking-Tsinghua Center for Life Sciences, Peking University, Beijing, China.

7. School of Public Health, Shandong University, Jinan, China.

8. Department of Obstetrics and Gynecology, University of Michigan, Ann Arbor, Michigan, United States of America.

9. Shandong Center for Disease Control and Prevention, Jinan, China

**Table of contents**

**Figure S1.** Number of differentially expressed genes and enrichment analysis

**Figure S2.** Effect of oocyte cryopreservation on the sex of blastocyst-stage embryos

**Figure S3.** X chromosome inactivation and *Xist* expression in embryonic cells

**Figure S4.** Cell cycle dynamics, and X-chromosome dosage in embryonic cells

**Table S1.** RT-qPCR primers used in the experiment

**Table S2.** Sample information and annotations

**Table S3.** Statistical table of differentially expressed genes in ICM between the Cryo and the Fresh groups

**Table S4.** Statistical table of differentially expressed genes in TE between the Cryo and the Fresh groups

**Table S5.** GO enrichment analysis statistics in the Fresh and the Cryo groups

**Table S6.** The proportion of different *Xist*-expressing cells across different cell cycles in males

**Figure S1.** Number of differentially expressed genes and enrichment analysis

**(A)** Quantitative comparison of differentially expressed genes (DEGs) between Fresh and Cryo groups across developmental stages. **(B)** Relative mRNA expression levels of *Bex1* and *Phlda2* in intact blastocysts derived from the Fresh and Cryo groups. Data are presented as mean ± SEM (n = 4). Statistical significance was determined using the two-tailed independent samples t-test. NS, *p* > .05. **(C)** Gene Ontology (GO) enrichment analysis of DEGs identified in blastocyst-stage embryos.

**Figure S2.** Effect of oocyte cryopreservation on the sex of blastocyst-stage embryos

**(A)** The ratio of expression level of X chromosome-specific genes to the total gene expression level. **(B)** The expression levels of Y chromosome-specific genes of all embryos. **(C)** Stacked bar chart depicting the counts and ratios of male and female embryos in the Fresh and Cryo groups. NS, *p* > .05).

**Figure S3.** X chromosome inactivation and *Xist* expression in embryonic cells

**(A)** Relative expression levels of X-linked genes in male TE and ICM cells from the Cryo group compared with the Fresh group. The red dashed line indicates a ratio of 1.0; NS, *p* > .05. **(B)** Violin plots showing the expression levels of *Xist*, *Atrx*, and *Spen* in TE and ICM in male cells of blastocysts. ***, *p* < .001; NS, *p* > .05 **(C)** Bar plots showing the proportions of cells with high and low *Xist* expression in TE and ICM cell populations of male blastocysts from the Cryo and Fresh groups. **(D)** The pseudo-time plot illustrates the developmental trajectory from MII oocytes to blastocyst stages, with the Fresh group shown on the left and the Cryo group on the right. Red dots represent cells with high *Xist* expression, while gray dots represent cells with low *Xist* expression. The progression from small to large dots indicates the developmental direction from early to late stages. Solid dots denote female samples, whereas transparent dots represent male or unspecified sex cells. **(E)** The pseudo-time plot illustrates the developmental trajectory from MII oocytes to blastocyst stages, with the Fresh group shown on the left and the Cryo group on the right. Red dots represent cells with high *Xist* expression, while gray dots represent cells with low *Xist* expression. The progression from small to large dots indicates the developmental direction from early to late stages. Solid dots denote male samples, whereas transparent dots represent female or unspecified sex cells.

**Figure S4.** Cell cycle dynamics, and X-chromosome dosage in embryonic cells

**(A)** Ridge plots comparing the overall expression levels and distribution of four canonical cell cycle-regulating genes (*Pcna*, *Top2a*, *Mcm6*, and *Mki67*) between the Fresh and Cryo groups across all embryonic cells. **(B)** Dimensionality reduction plots visualizing cell cycle phase progression (G1, S, or G2/M) specifically in female embryonic cells, display detailed phase distributions across different cell types in both the Fresh and Cryo groups. **(C)** Dimensionality reduction plots highlighting the distribution of *Xist*-highly expressing cells (marked in red) specifically within male blastocysts. Cell types are indicated by distinct shapes (circles for TE, triangles for ICM), and the plots are split by the Fresh (left) and Cryo (right) groups. **(D)** Dot plots illustrating the cell cycle phase distribution patterns of *Xist*-high (red) and *Xist*-low (grey) cells within the TE and ICM subpopulations of male blastocysts. The size of the dots represents the percentage of cells at the specified cell cycle stage. **(E)** Cells are colored according to their module scores (blue gradient: low to high). The upper panel shows the cell cycle score, and the lower panel shows the X-chromosome score.

**Table S1.** RT-qPCR primers used in the experiment

| **Gene** | **Primer** | RNA Sequence (5’→3’sense) |
| --- | --- | --- |
| *Bex1* | Forward Primer | AGCAGGTCTGAGAAGCAGAAG |
|  | Reverse Primer | GATCTTTGGACTCCATTACTCCTGG |
| *Phlda2* | Forward Primer | CGCCAAGGAGCTGTTTTTCC |
|  | Reverse Primer | TAGCGGGGAAAGTCTTGCAG |
| *Rn18S* | Forward Primer | CGCCGCTAGAGGTGAAATTCT |
|  | Reverse Primer | CGAACCTCCGACTTTCGTTCT |

**Table S6** The proportion of different *Xist*-expressing cells across different cell cycles in males

| **Cell_type** | **Group** | ***Xist*_expression** | **Phase** | **Cell_Num (%)** | ***P*** |
| --- | --- | --- | --- | --- | --- |
| TE | Fresh | *Xist*_Higher | G1 | 4 (26.67%) | *p* > .05 |
|  |  | *Xist*_Higher | G2M | 4 (26.67%) |  |
|  |  | *Xist*_Higher | S | 7 (46.67%) |  |
|  |  | *Xist*_Lower | G1 | 6 (13.33%) |  |
|  |  | *Xist*_Lower | G2M | 21 (46.67%) |  |
|  |  | *Xist*_Lower | S | 18 (40.00%) |  |
|  | Cryo | *Xist*_Higher | G1 | 2 (7.14%) |  |
|  |  | *Xist*_Higher | G2M | 15 (53.57%) |  |
|  |  | *Xist*_Higher | S | 11 (39.29%) |  |
|  |  | *Xist*_Lower | G1 | 14 (13.59%) |  |
|  |  | *Xist*_Lower | G2M | 51 (49.51%) |  |
|  |  | *Xist*_Lower | S | 38 (36.89%) |  |
| ICM | Fresh | *Xist*_Higher | G1 | 1 (5.00%) | *p* >.05 |
|  |  | *Xist*_Higher | G2M | 15 (75.00%) |  |
|  |  | *Xist*_Higher | S | 4 (20.00%) |  |
|  |  | *Xist*_Lower | G1 | 6 (12.77%) |  |
|  |  | *Xist*_Lower | G2M | 31 (65.96%)) |  |
|  |  | *Xist*_Lower | S | 10 (21.28%) |  |
|  | Cryo | *Xist*_Higher | G1 | 1 (9.09%) |  |
|  |  | *Xist*_Higher | G2M | 6 (54.55%) |  |
|  |  | *Xist*_Higher | S | 4 (36.36%) |  |
|  |  | *Xist*_Lower | G1 | 6 (8.00%) |  |
|  |  | *Xist*_Lower | G2M | 39 (52.00%) |  |
|  |  | *Xist*_Lower | S | 30 (40.00%) |  |
